# Flexible Neural Network Realized by the Probabilistic SiO*_x_* Memristive Synaptic Array for Energy‐Efficient Image Learning

**DOI:** 10.1002/advs.202104773

**Published:** 2022-02-16

**Authors:** Sanghyeon Choi, Jingon Jang, Min Seob Kim, Nam Dong Kim, Jeehyun Kwag, Gunuk Wang

**Affiliations:** ^1^ KU‐KIST Graduate School of Converging Science and Technology Korea University 145 Anam‐ro, Seongbuk‐gu Seoul 02841 Republic of Korea; ^2^ Institute of Advanced Composite Materials Korea Institute of Science and Technology 92 Chudong‐ro, Bongdong‐eup Wanju‐gun Jeollabuk‐do 55324 Republic of Korea; ^3^ Department of Brain and Cognitive Engineering Korea University Seoul 02841 Republic of Korea; ^4^ Department of Integrative Energy Engineering Korea University 145 Anam‐ro, Seongbuk‐gu Seoul 02841 Republic of Korea

**Keywords:** barristor, drop‐connected network, neuromorphic computing, probabilistic synapse, silicon, silicon oxide

## Abstract

The human brain's neural networks are sparsely connected via tunable and probabilistic synapses, which may be essential for performing energy‐efficient cognitive and intellectual functions. In this sense, the implementation of a flexible neural network with probabilistic synapses is a first step toward realizing the ultimate energy‐efficient computing framework. Here, inspired by the efficient threshold‐tunable and probabilistic rod‐to‐rod bipolar synapses in the human visual system, a 16 × 16 crossbar array comprising the vertical form of gate‐tunable probabilistic SiO*_x_* memristive synaptic barristor utilizing the Si/graphene heterojunction is designed and fabricated. Controllable stochastic switching dynamics in this array are achieved via various input voltage pulse schemes. In particular, the threshold tunability via electrostatic gating enables the efficient in situ alteration of the probabilistic switching activation (*P*_*Act*_) from 0 to 1.0, and can even modulate the degree of the *P*_*Act*_ change. A drop‐connected algorithm based on the *P*_*Act*_ is constructed and used to successfully classify the shapes of several fashion items. The suggested approach can decrease the learning energy by up to ≈2,116 times relative to that of the conventional all‐to‐all connected network while exhibiting a high recognition accuracy of ≈93 %.

## Graphical abstract

Sparse activity and connectivity via probabilistic synapses have received considerable attention because they can facilitate the energy‐efficient functionalities in the human brain. Inspired by the human visual system, a gate‐tunable and probabilistic SiO*_x_* memristive synaptic barristor crossbar array is fabricated and utilized as a flexible neural network that can actively mimic the sparse neural network.

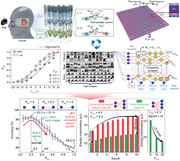

## 1 Introduction

The complex neural circuits of the human brain, comprising 10^11^ neurons connected via 10^15^ synapses, efficiently process a vast amount of information, including cognitive functions and memory.^[^ ^1^, ^2^ ^]^ Neurons generate electrical signals called spikes, which trigger the release of neurotransmitters from synapses, thus forming the basis of neural information processing in the brain.^[^ ^1^, ^2^ ^]^ In a biological neural networks, the neurons are sparsely connected with probabilistic synaptic connectivity.^[^ ^3^, ^4^, ^5^, ^6^, ^7^, ^8^, ^9^ ^]^ For example, the connection probability of cortical neurons has been experimentally determined to be ≈10 %,^[^ ^3^, ^4^ ^]^ and the synapses have probabilistically low neurotransmitter release.^[^ ^5^, ^6^ ^]^ Moreover, only a few neurons (<0.5 %) in the visual cortex respond to natural images, representing sensory information with a sparse spike.^[^ ^7^, ^8^, ^9^ ^]^ Such sparse activity in probabilistic synapses is regarded as a general feature of the brain's neural network, enabling extremely low‐power and fault‐tolerant computations (i.e., ≈1 fJ per synaptic activity) against diverse unstructured cognitive tasks.^[^ ^3^, ^4^, ^5^, ^6^, ^7^, ^8^, ^9^ ^]^ Due to this extremely high energy efficiency, a neuromorphic hardware system mimicking the key principles of the human brain has been proposed for sustainable computing technology in the big data era.^[^ ^10^, ^11^, ^12^, ^13^, ^14^, ^15^, ^16^ ^]^

Generally, a conventional artificial neural network (ANN) for neuromorphic computing is based on an all‐to‐all connected feed‐forward neural network comprised of several hidden interlayers, where each layer is individually connected via deterministic and analog synaptic cells.^[^ ^10^, ^11^, ^12^, ^13^, ^14^, ^15^, ^16^ ^]^ Although this network offers advantages such as superior learning ability and high cognitive accuracy for a given task, it inevitably requires massive repetitive calculations for synaptic weight updates during the learning process, thus leading to high power consumption and the need for complex electrical circuitry. In addition, since this network structure is fixed and inflexible toward further changes, its ability to efficiently handle a range of tasks is limited, unlike the brain's flexible neural network. Hence, the challenges presented by such complex, power‐hungry, and nonflexible neural network architectures must be overcome in order to achieve low‐power ubiquitous artificial intelligence in a miniaturized and distributed edge computing system.

Recently, the memristor has been highlighted as a promising artificial synaptic device node for the realization of neuromorphic hardware systems due to the simple device structure and excellent synaptic plasticity, along with low power and a nanosecond switching speed.^[^ ^11^, ^12^, ^13^, ^14^, ^15^, ^16^ ^]^ At the same time, there is a well‐known challenge that the inherent switching stochasticity of the memristor must be overcome as this property might interrupt the correct updating of the synaptic weight during the learning process and significantly increase the time taken to find the optimal weight values.^[^ ^11^, ^12^, ^13^ ^]^ In this sense, many memristor studies have focused on optimizing the device structure and operation scheme in order to realize a deterministic switching transition with low variation for the high‐precision cognitive capability of the neuromorphic hardware system.^[^ ^14^, ^15^, ^16^, ^17^, ^18^, ^19^ ^]^ For example, Prezioso et al. have optimized the composition and thickness of the TiO_2_ memristor by alternating various experimental conditions for low device variability, and demonstrated the correct recognition of simple alphabet images.^[^ ^14^ ^]^ Boybat et al. suggested a global counter‐based arbitration scheme with multiple memristors (*N*) per synapse, capable of decreasing the variation in the updating of the synaptic weight by$\;\sqrt{N}$, thereby improving the recognition accuracy of handwritten digit patterns.^[^ ^15^ ^]^ Moreover, Gao et al. reported a three‐dimensional structure consisting of several parallel memristors on the same nanopillar in order to decrease the resistance variation, which can improve the pattern recognition performance.^[^ ^16^ ^]^

By contrast, in pursuit of comparable efficiency to that of the human brain, a few studies on the device implementation of probabilistic artificial synapses and their applications have been reported to date.^[^ ^20^, ^21^ ^]^ For example, Serb et al. fabricated a Pt/TiO_2_/Pt memristor capable of encoding conditional probabilities as a form of the resistive state, and proposed a probabilistic neural network based on this memristor for recognizing simple binary patterns.^[^ ^20^ ^]^ Dalgaty et al. implemented the malignant tissue recognition, heart arrhythmia detection, and cartpole reinforcement learning task based on the intrinsic variation of a TiN/HfO_2_/Ti/TiN crossbar array.^[^ ^21^ ^]^ These studies are noteworthy in terms of the implementation of probabilistic memristor synapses and their possible neuromorphic computing applications. However, these neural network structures remain limited by the intrinsic variability of the used memristor and cannot provide a flexible neural network with controllable variability for the handling of various probabilistic ANNs and datasets. Moreover, these proposed probabilistic neural networks do not reflect sparse features of the human brain, such as the dynamic randomness of the network configuration and the irregular activation rate between different cells. In this sense, the development of a controllable probabilistic artificial synapse equipped with high ON‐OFF ratio and operational stability, and a flexible neural network with an optimized learning algorithm that can mimic the sparse activity of the biological cortex network is highly demanded.

Inspired by the probabilistic rod‐to‐rod bipolar synapses of the visual cortex system, a gate‐tunable and probabilistic artificial synaptic array is fabricated by employing vertically integrated SiO*_x_* barristor synapses for the flexible neural network. The probabilistic activation (*P*_*Act*_) of the synaptic array can be nonlinearly controlled from 0 to 1.0 via electrostatic gating, and its degree is also widely modulated by employing various electrical parameters, thus exhibiting several sigmoidal forms. This array mimics the stochastic dynamics of the synaptic signaling in a sparse neural network, and is capable of a low power learning process. With this result, a drop‐connected network is constructed that reflects the sparse connectivity of biological neural networks without the need for complex learning processes. Further, the energy consumption and recognition accuracy of the suggested drop‐connected network for several fashion‐item images are evaluated based on the probabilistic degree of SiO*_x_* synaptic activity.

## 2 Results and Discussion

**Figure 1:**
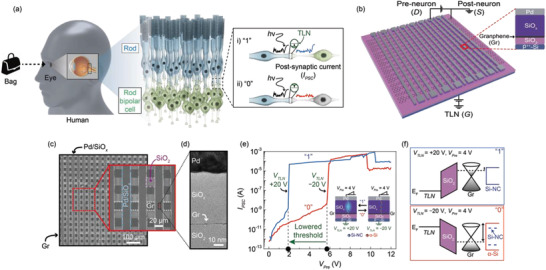
The rod‐to‐rod bipolar synaptic network in the human visual system and the gate‐tunable and probabilistic SiO*_x_* synaptic barristor crossbar array. a) Schematic diagrams of the human visual system during image recognition, in which individual synapses are formed between the rods and rod bipolar cells. The right box shows the light‐driven *I*_*PSC*_ signal transmitted through the synapse for the “1” and “0” states. The shift in the TLN criterion (the green line) signifies the threshold shift, distinguishing the light‐driven response (“1” state) from noise (“0” state). b) A schematic diagram of a 16 × 16 crossbar array consisting of the gate‐tunable and probabilistic SiO*_x_* synaptic barristor cells. The top Pd and bottom Gr lines are defined as the pre‐ and post‐neurons corresponding to the top rod and bottom rod bipolar cell, respectively. The bottom gate is defined as a TLN, which can determine the threshold for signal transmission. The inset shows an enlarged schematic diagram of the Pd/SiO*_x_*/Gr/SiO2/p++‐Si junction structure. c) A top‐view SEM image of the fabricated array device with a line width of 20 µm. d) A cross‐sectional HR‐TEM image of the device at the crosspoint in the array. e) Exemplary *I*_*PSC*_–*V*_*Pre*_ curves for the “1” and “0” states obtained at *V*_*TLN*_ = −20 (red) and +20 V (blue). The insets illustrate the junction schematics for the “1” and “0” states where the formation of Si phase filament is localized at the SiO*_x_* edge. Note that Si‐NC and *α*‐Si are represented as blue and red circles, respectively. f) The corresponding energy band diagrams for the SiO*_x_* synaptic barristor at *V*_*Pre*_ = 4 V with respect to *V*_*TLN*_ = +20 V (upper, “1”) and −20 V (lower, “0”).

**Figure** **1**a illustrates the transmission of signals from the rods to the rod bipolar cells of the human visual system while recognizing a bag image as an example. It is known that the human eye can control retinal sensitivity to allow the detection of a tiny flash of light, even in the dark; that is, it can gradually adapt to a dark environment to enable the recognition of the shapes of objects.^[^ ^22^, ^23^ ^]^ In the retinal signal processing system, a threshold‐like nonlinearity (TLN) in the rod‐to‐rod bipolar synapses can distinctly facilitate separation between the light‐driven response and synaptic noise.^[^ ^22^, ^23^ ^]^ In particular, TLN can act as a threshold gate for the binary states (“1” or “0” state). It sets an amplitude criterion (the threshold) to determine whether the input signals can be delivered in the form of post‐synaptic current (*I*_*PSC*_) to the rod photoreceptor (the inset of Figure 1a). Hence, only a light‐driven response that exceeds the threshold value can be transmitted to the next rod bipolar cell. Interestingly, the threshold can be modified by the degree of signaling cascade through G‐protein in the post‐synaptic region,^[^ ^22^, ^23^ ^]^ which is denoted by the shift of the green lines in the inset of Figure 1a. Therefore, the signal transmitted at the rod‐to‐rod bipolar synapse can be modulated by both the degree of threshold shift and the magnitude of the light‐driven responses. This indicates that the transmission probability through the rod bipolar cell is controllable, thus enabling the efficient conveyance of small signals to higher visual centers in order to accelerate the image recognition process.^[^ ^5^, ^6^, ^7^, ^22^, ^23^ ^]^

Inspired by the efficient threshold‐tunable signal transmission in the binary form at the rod‐to‐rod bipolar synapse, we devised a tunable and probabilsitic artificial synaptic crossbar array based on a vertically integrated SiO*_x_* memristor with a bottom graphene barristor (Figure 1b). Figure 1b illustrates a gate‐tunable and probabilistic SiO*_x_* synaptic barristor crossbar array, which consists of a vertically stacked Pd/SiO*_x_*/graphene (Gr)/SiO_2_/P^++^‐Si structure (the inset). In this device structure, the Gr barristor part can additionally regulate the interfacial Schottky barrier located at the interface between the Si phase in SiO*_x_* edge and the Gr to actively control the stochastic formation of the Si phase filament in the SiO*_x_* memristor, which will be further discussed in the following results. Here, we define the top Pd (drain, *D*), the bottom Gr (source, *S*), and the bottom highly p‐doped Si (gate, *G*) as pre‐neuron, post‐neuron, and TLN, respectively. Figure 1c shows a top‐view scanning electron microscopy (SEM) image of the fabricated 16 × 16 probabilistic SiO*_x_* synaptic barristor crossbar array, where the enlargement reveals a cell line width of 20 μm. Figure 1d shows a cross‐sectional high‐resolution transmission electron microscopy (HR‐TEM) image of the probabilistic SiO*_x_* synaptic barristor located at the crosspoint of the array. These results indicate that the vertical channel junction is maintained in spite of the three‐terminal structure, thus leading to a simple and highly dense integration similar to that of a two‐terminal memristor (i.e., 4F^2^ footprint), which is further illustrated and described in Figure S1 (Supporting Information). The detailed fabrication processes are presented in the Experimental Section.

In a memristor cell, the SiO*_x_* layer enables a unipolar switching function driven by two different Si phases (i.e., a semi‐metallic Si nanocrystal (Si‐NC) phase and an insulating amorphous Si (α‐Si) phase) localized at the SiO*_x_* edge after the completion of the electroforming process (Figure S2, Supporting Information).^[^ ^24^, ^25^, ^26^, ^27^, ^28^, ^29^, ^30^, ^31^ ^]^ It is known that the Si phase transition occurs stochastically during the repeated switching cycles depending on the device structure and operating variables.^[^ ^25^, ^27^, ^28^, ^29^, ^30^, ^31^ ^]^ Previously, such an unipolar SiO*_x_* (1 < *x* < 2) memory operated by the formation of a conducting Si‐NC filament (sub‐5 nm) and various device architectures with high ON‐OFF ratio (>10^5^) and fast switching speed (≈10 ns) had been already demonstrated.^[^ ^24^, ^25^, ^26^, ^27^, ^28^, ^29^, ^30^, ^31^ ^]^ We have also demonstrated the unipolar SiO*_x_* switching behaviors based on various junction structures such as well‐defined single^[^ ^27^ ^]^ or multiple nanopores,^[^ ^28^ ^]^ nanorods,^[^ ^29^ ^]^ nanogaps,^[^ ^27^, ^29^ ^]^ two‐terminal crossbar array,^[^ ^30^ ^]^ and three‐terminal junction architecture for implementation of logic gates and memory applications.^[^ ^31^ ^]^ With these results, it is expected that the SiO*_x_* memristive synaptic barristor will be capable of scaling to nanoscale, taking into account the feasibility of nanoscale Si‐NC filament.^[^ ^27^, ^29^ ^]^

Recently, there have been several attempts to apply the SiO*_x_* memristor as an artificial synaptic node for a neuromorphic computing system.^[^ ^32^, ^33^ ^]^ For example, Chang et al. suggested a SiO*_x_* memristor as an artificial synapse and investigated the essential synaptic functionalities such as long‐term plasticity and spiking‐timing‐dependent‐plasticity (STDP) for neuromorphic hardware systems.^[^ ^32^ ^]^ Similarly, Zarudnyi et al. implemented the STDP synaptic behaviors by using a SiO*_x_* memristor consisting of TiN/SiO*_x_*/TiN junction structure in order to simplify the hardware design of neuromorphic computing applications.^[^ ^33^ ^]^ However, these types of two‐terminal SiO*_x_* memristor structures are not appropriate for actively controlling the dynamic stochasticity of the Si‐NC filament and cannot be applied as a synaptic node for a flexible neural network. In this sense, our suggested gate‐tunable and probabilistic SiO*_x_* synaptic array enabling flexible neural networks can be differentiated from the prior SiO*_x_* and other types of metal‐oxide memristor structures.

In this integrated SiO*_x_* memristor with bottom graphene barristor, the Si phases formed at the SiO*_x_* edge lead to the barristor configuration together with the bottom Gr.^[^ ^31^, ^34^ ^]^ This barristor can modulate the Schottky barrier height at the Si phase filament/Gr interface via the electrostatic gating, thus enabling electrical regulation of the charge transport within the entire device. This active tuning can enable the gate to act as the TLN in a rod‐to‐rod bipolar synapse. In other words, the gating is able to establish and shift the threshold, enabling changes in the *I*_*PSC*_ even when a small electrical input is applied. For example, as shown in Figure 1e, different current–voltage (*I*_*PSC*_–*V*_*Pre*_) switching curves can be obtained by sweeping *V*_*Pre*_ from 0 to 12 V with respect to various *V*_*TLN*_ values. The lowered threshold is clearly observed as the *V*_*TLN*_ increases from −20 to +20 V, thus switching the *I*_*PSC*_ from “0” (OFF) state into “1” (ON) state at the same *V*_*Pre*_. A higher *V*_*TLN*_ = 20 V can decrease in the Schottky barrier at the Si phase/graphene interface, resulting in the transition from the α‐Si to Si‐NC phase at relatively low *V*_*Pre*_ (1.9 V, threshold voltage). Namely, the “1” state can be made at relatively low *V*_*Pre*_. However, at *V*_*TLN*_ = −20 V, the Schottky barrier is increased, then the required *V*_*Pre*_ (threshold voltage) for SET is increased up to 5.7 V. Therefore, the device did not switch to “1” state below the *V*_*Pre*_ = 5.7 V (i.e., sustaining “0” state). This result corresponds to the TLN in the rod‐to‐rod bipolar synapse, considering that a higher *I*_*PSC*_ can be generated even with a relatively small electrical input (the inset of Figure 1a,e). Figure 1f explains the *V*_*TLN*_‐dependent switching mechanism by the energy band alignment. It is known that the bandgap of the α‐Si from ≈1.6–1.8 eV (the red line of Figure 1f), whereas that of Si‐NC is less than ≈1.0 eV (the blue (dotted) line of Figure 1f).^[^ ^35^, ^36^ ^]^ In our device structure, the Si phase filament consisting of two Si phase (Si‐NC and α‐Si) at the SiO*_x_* edge is formed on the graphene after completion of the electroforming process (Figure S2, Supporting Information) and acts as semiconducting layer for the graphene barristor. Because the electrostatic gating can shift the Fermi level (E_F_) of graphene,^[^ ^37^ ^]^ the barrier height at the Si phases/graphene interface can be dependent on the *V*_*TLN*_. As the *V*_*TLN*_ increased, the E_F_ of the graphene approached the conduction band of the Si phases with the increase of the electron concentration in the graphene. These results lead to lowering the Schottky barrier height, increasing the effective electric field applied across the Si phase filament at the same *V*_*Pre*_. As a result, the transition from α‐Si to Si‐NC at the SiO*_x_* edge can be possible at lower SET (threshold) voltage. We also quantitatively estimated the modulation of the Schottky barrier height (*Φ*_*B*_) at the Si phases/Gr interface at *V*_*Pre*_ = 1 V based on the *V*_*TLN*_ (Figures S3, S4, and Table S1, Supporting Information). It was found that the *Φ*_*B*_ at the Si phases/Gr interface is decreased from 0.82 to 0.45 eV when increasing *V*_*TLN*_ from −20 to +20 V.

**Figure 2:**
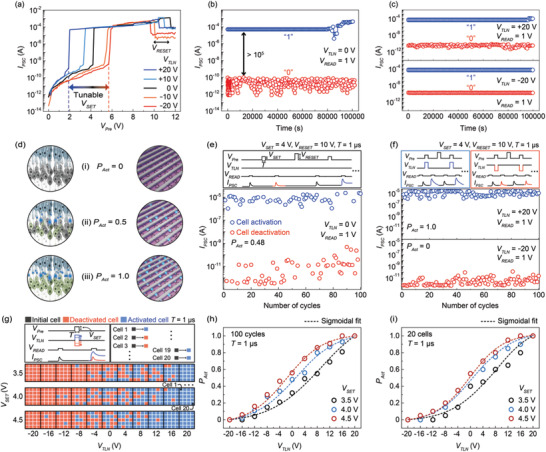
Gate‐tunable switching and probabilistic characteristics. a) The *I*_*PSC*_–*V*_*Pre*_ switching curves of the gate‐tunable and probabilistic SiO*_x_* synaptic barristor at various *V*_*TLN*_ values ranging from −20 to +20 V. We observed the *V*_*TLN*_‐dependent *V*_*SET*_ values (color‐graded arrow). *V*_*RESET*_ is independent on the *V*_*TLN*_ value (black arrow) due to the Joule heating‐driven process. Retention capabilities of the device at b) *V*_*TLN*_ = 0 and c) ±20 V. The *I*_*PSC*_ values corresponding to “0” and “1” states were consecutively measured at *V*_*READ*_ = 1 V for 10^5^ s with ∆*t* = 1 s after the completion of the SET‐ and RESET‐switching transition. d) Schematic diagrams of the rod‐to‐rod bipolar synaptic network (the left) and the probabilistic SiO*_x_* synaptic barristor crossbar array (the right) at various *P*_*Act*_ values. Plots of the *I*_*PSC*_ values at *V*_*READ*_ = 1 V after application of a *V*_*SET*_ pulse during 100 consecutive cycles at various *V*_*TLN*_ values. As shown in the top panels, one cycle consisted of *V*_*READ*_ = 1 V, *V*_*SET*_= 4 V, *V*_*READ*_ = 1 V, and *V*_*RESET*_ = 10 V for *T* = 1 µs, and *V*_*TLN*_ was set to e) 0 V and f) ±20 V. g) Matrix visualization of the activation and deactivation of the selected 20 cells after the application of various voltage pulse schemes (*V*_*SET*_ and *V*_*TLN*_). The activated and deactivated cells are represented as blue and red boxes, respectively. As shown in the top panels, the initial and final *I*_*PSC*_ values determine the cell activation and deactivation. The *V*_*SET*_ pulse was varied from 3.5 to 4.5 V, and *V*_*TLN*_ pulse was varied from −20 to +20 V. Plots of *P*_*Act*_ values at *V*_*SET*_ = 3.5, 4.0, and 4.5 V for *T* = 1 µs as a function of *V*_*TLN*_ from −20 to 20 V with respect to h) 100 cycles and i) 20 cells. All the plots follow exponential probability distributions following the sigmoidal curves (dotted lines).

**Figure** **2**a shows representative *I*_*PSC*_–*V*_*Pre*_ switching curves of the fabricated gate‐tunable SiO*_x_* synaptic barristor obtained at various *V*_*TLN*_ values in the range of −20 to +20 V. Here, typical unipolar switching behaviors are observed regardless of the *V*_*TLN*_ value. In other words, a sudden increase (SET) and decrease (RESET) in the *I*_*PSC*_ can be achieved in the same voltage polarity. Note that the *V*_*Pre*_ values required for the SET– and RESET–switching transition correspond to *V*_*SET*_ and *V*_*RESET*_, respectively. In particular, *V*_*SET*_ can be significantly decreased from 5.7 to 1.9 V by increasing *V*_*TLN*_ from −20 to +20 V (Figure 2a). This can be explained by the controllable Schottky barrier height at the Si phase/Gr interface according to *V*_*TLN*_ value, which alters the effective electric field applied across the Si phase filament at the same *V*_*Pre*_; hence, the crystallization rate of α‐Si can be determined by the *V*_*TLN*_ value.^[^ ^25^, ^27^, ^29^ ^]^ However, the *V*_*RESET*_ values are seen to vary irregularly between 9.5 and 11.5 V regardless of *V*_*TLN*_ values (Figure 2a). In other words, the *V*_*RESET*_ value is independent of the *V*_*TLN*_ and is therefore not tunable. This is because the RESET switching process is associated with the filament rupture via the Joule heating effect.^[^ ^25^, ^27^, ^29^ ^]^ Note that programming voltages can be further reduced through a variety of feasible means, including the incorporation of metal interlayers within SiO*_x_* layers^[^ ^38^ ^]^ and the design of the operating scheme.^[^ ^28^ ^]^ In addition, the *V*_*TLN*_ value can be effectively lowered if the 285 nm‐thick SiO_2_ layer is replaced with a thinner oxide or high‐k dielectric layer.^[^ ^39^ ^]^ As a result, at the same *V*_*TLN*_, the *V*_*SET*_ of the SiO*_x_* memristive synaptic barristor could be lowered even further. Figures 2b,c show the retention properties of “1” and “0” states by the fabricated synaptic cell as a function of time for *V*_*TLN*_ values of 0 and ± 20 V, respectively. Here, the “1” and “0” states are retained for 10^5^ s, with a ratio of more than 10^5^, regardless of the *V*_*TLN*_. For this experiment, the read voltage (*V*_*READ*_) was set to 1 V and did not alter the conductance states of the device under the applied *V*_*TLN*_ regime, as shown in Figure S5 (Supporting Information). These results verify that the proposed device structure can learn and store the updated synaptic weights after the completion of the learning process. Note that the observed variation in “0” state might be ascribed to the random distribution of α‐Si phases in the middle of the Si‐NC conductive filament; i.e., an incomplete RESET switching transition.

In the visual system, the signal can be transmitted through the rod‐to‐rod bipolar synapses depending on a tunable probabilistic activation (*P*_*Act*_) (the left of Figure 2d). For example, a *P*_*Act*_ value of 1.0 indicates that the rod‐to‐rod bipolar synapses are entirely activated, while a *P*_*Act*_ value of 0 indicates that they are entirely deactivated, in response to a given input. If half of the bipolar synapses are randomly activated, however, a *P*_*Act*_ value of 0.5 will be obtained. The gate‐tunable SiO*_x_* synaptic barristor itself can mimic these *P*_*Act*_ and TLN functions, and its array structure can also mimic the controllable sparse activation in the visual cortex neural network (the right of Figure 2d). To investigate the probabilistic activation of the gate‐tunable SiO*_x_* synaptic barristor, the *P*_*Act*_ of the fabricated synaptic cells in the array were statistically evaluated according to various programming input schemes (Figure 2e–i). Figure 2e shows the evolution of *I*_*PSC*_ values at *V*_*READ*_ = 1 V after the application of a *V*_*SET*_ pulse during 100 cycles. Here, one cycle consists of *V*_*READ*_ = 1 V, *V*_*SET*_ = 4 V, *V*_*READ*_ = 1 V, and *V*_*RESET*_ = 10 V for *T* = 1 µs, and *V*_*RESET*_ is used to initialize the cell in the “0” state (the top panel of Figure 2e). If *I*_*PSC*_ remains almost unchanged after the application of a *V*_*SET*_ pulse (i.e., “0” → “0”), the cell is considered to be deactivated (red circles). However, if *I*_*PSC*_ significantly increases by at least 10^5^ after application of a *V*_*SET*_ pulse (i.e., “0” → “1”), the cell is considered to be activated (blue circles). As shown in Figure 2e, despite the application of the same *V*_*SET*_ pulses, the number of cell activation is observed to be 48 times out of 100 consecutive cycles, giving *P*_*Act*_ = 0.48. Such a *V*_*SET*_ pulse‐dependent *P*_*Act*_ could be implemented on the conventional two‐terminal SiO*_x_* memristors (i.e., *V*_*TLN*_ = 0 V) as well. However, the proposed device structure also allows *P*_*Act*_ to be efficiently controlled at the same *V*_*SET*_ pulses via the introduction of the *V*_*TLN*_. This is because the alteration in the effective electric field via gating can regulate the stochastic phase transition from α‐Si to Si‐NC via shifting of the *V*_*SET*_. As shown in the top of Figure 2f, when *V*_*TLN*_ increases to +20 V, the cells are activated 100% during 100 cycles, i.e., *P*_*Act*_ = 1.0. Conversely, when *V*_*TLN*_ decreases to −20 V, the cells are deactivated 100% during 100 cycles, i.e., *P*_*Act*_ = 0, as shown in the bottom of Figure 2f. Thus, *V*_*TLN*_ plays a similar role to that of the threshold shift of the rod‐to‐rod bipolar synapse in controlling the cell activation stochasticity. Note that the corresponding *I*_*PSC*_ behaviors of the selected synaptic cell according to the various *V*_*TLN*_ values are presented in Figure S6 (Supporting Information). Moreover, the SiO*_x_* memristive synaptic barristor has an acceptable stability regarding the consecutive stochastic transition between Si‐NC and α‐Si phases (see Figure S7, Supporting Information). Meanwhile, Figure 2g visualizes the activation (blue boxes) or deactivation (red boxes) of the selected 20 cells in the array as a function of *V*_*TLN*_ for various *V*_*SET*_ pulses at *T* = 1 µs. Here, the activation probability (*P*_*Act*_) of the 20 cells can be estimated by the proportion of blue boxes, and ranges from 0 to 1.0. The number of blue boxes (and, hence, the *P*_*Act*_) is increased as the *V*_*SET*_ and *V*_*TLN*_ are each increased. Similarly to the results in Figure 2f, all 20 cells are deactivated (*P*_*Act*_ = 0) when the *V*_*TLN*_ is set to −20 V, and all 20 cells are activated (*P*_*Act*_ = 1.0) when *V*_*TLN*_ is set to +20 V, regardless of the applied *V*_*SET*_.

Figures 2h,i show the statistical evolution of *P*_*Act*_ behaviors as a function of the *V*_*TLN*_ under various *V*_*SET*_ values at *T* = 1 µs during 100 consecutive cycles for one cell (Figure 2h) and for 20 cells (Figure 2i), respectively. All the *P*_*Act*_ behaviors are well‐fitted by the sigmoidal curves, and are mathematically expressed as *P*_*Act*_ = *a* / (1 + exp(−*b* × (*V*_*TLN*_‐*c*))), where the fitting parameters *a*, *b*, and *c* are presented in Table S2 (Supporting Information). As the sigmoid is considered as a natural stochastic function in the field of the probabilistic neural network and machine learning,^[^ ^40^ ^]^ the *P*_*Act*_ of the gate‐tunable and probabilistic SiO*_x_* synaptic barristor crossbar array would be available for a flexible neural network. The detailed *P*_*Act*_ values are all enumerated in Table S3 (Supporting Information). In addition to *V*_*TLN*_, electrical parameters such as *V*_*SET*_ and *T* can be used to adjust the *P*_*Act*_ (Figure S8 and Table S4, Supporting Information). We should note here that a fast and low‐energy operation could be achieved by combining a shorter *T* and lower *V*_*SET*_ with the appropriate *V*_*TLN*_ value. Note that the minimum *T* for the cell activation is found to ≈35 ns (Figure S9, Supporting Information). These statistical investigations thus confirm that each synaptic cell in the array can exhibit a well‐defined and tunable *P*_*Act*_ nature.

**Figure 3:**
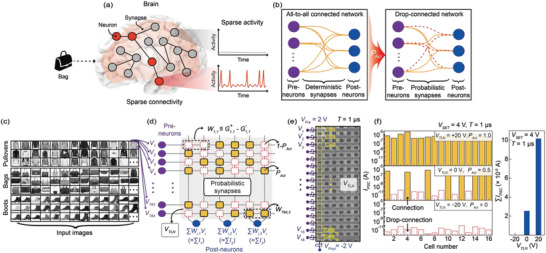
The drop‐connected neural network. a) A schematic diagram of the sparsely connected neural network consisting of activated neurons (red circles), deactivated neurons (gray circles), and synapses (lines) in the human brain during image learning and recognition, along with the sparse activity (the right inset). b) Schematic diagrams of the single‐layer network for the conventional all‐to‐all connected and drop‐connected neural networks comprised of deterministic and probabilistic synapses, respectively. *P*_*Act*_ indicates the switching‐transition probability of the synaptic cells for the drop‐connected network. c) Examples of the fashion‐item datasets (Pullovers, Bags, and Boots). Each fashion item has 6,000 and 1,000 different image datasets for the learning and inference stages, respectively. d) A flow diagram illustrating the recognition process based on the drop‐connected network comprising 784 pre‐neurons (purple circles) and 3 post‐neurons (blue circles) with 2,352 synaptic weights (rectangles). The grey region represents *V*_*TLN*_, enabling the efficient modulation of *P*_*Act*_. e) The used *V*_*SET*_ programming scheme in the array for the drop‐connected network configuration. The yellow dotted boxes indicate the programmed 16 cells of the selected column line. Note that *V*_*Pre*_ and *V*_*Post*_ were set to 2 and −2 V (i.e., *V*_*SET*_ = 4 V) with *T* = 1 µs for various *V*_*TLN*_ values. f) Histogram plots for the *I_PSC_* values of the selected 16 cells after application of the *V*_*SET*_ pulses. The *I*_*PSC*_ values were measured at *V*_*READ*_ = 1 V. The orange and red dotted boxes indicate the connection and drop‐connection, respectively, after the completion of *V*_*SET*_ programming. A higher *V*_*TLN*_ can lead to the more the connection of the selected 16 cells due to the increased *P*_*Act*_. The right inset shows the changeable ∑*I*_*PSC*_ values according to *V*_*TLN*_, indicating the flexibility of the weight updates based on the *V*_*TLN*_‐dependent *P*_*Act*_ behaviors.

**Figure** **3**a illustrates a sparsely connected neural network in the human brain during the process of image learning and recognition. Here, the red circles represent activated neurons, which can only communicate with one another via the synapses (solid lines), while the gray circles represent deactivated neurons. As indicated by the right inset of Figure 3a, this sparse connectivity and activity is inevitably generated by the probabilistic operation of the synapse, and effectively reduces the generation of electrical spikes in the neurons across the network, thus enabling low‐power neural processing during the cognitive task. To apply the gate‐tunable and probabilistic SiO*_x_* memristive synaptic barristor crossbar array to a sparsely connected neural network, we designed a drop‐connected neural network connected by probabilistic synapses that can be operated based on *P*_*Act*_ (Figure 3b).^[^ ^41^ ^]^ In this flexible neural network, certain synaptic weights are randomly selected and updated by designating a probability *P*_*Act*_ (orange lines), while the drop‐connection occurs at 1*−P*_*Act*_ (red dotted lines). Thus, certain synaptic connections represented by the red dotted lines are excluded to ensure that they are not updated during the learning stage. The suggested drop‐connected network, which can intentionally be controlled to a specific *P*_*Act*_, readily allows random selection of the activated cell by programming the voltage alone, thus effectively enabling a power‐saving learning process without requiring additional electrical circuitry and computation power. Moreover, because the network configuration with *P*_*Act*_ can be iteratively reshaped during the learning process, the ensemble effect of learning variables driven by different sub‐networks can be simply considered in the drop‐connected neural network, facilitating fault‐tolerant inference (Figure S10, Supporting Information).^[^ ^42^ ^]^ In addition, co‐adaptation, which is mostly determined by certain abnormal synaptic weights in a network, can be effectively prevented while learning images (Figure S10, Supporting Information).^[^ ^43^ ^]^ Consequently, the important aspects of the sparse neural network driven by the probabilistic activity of biological synapses can be emulated by this drop‐connected network configuration with *P*_*Act*_. We should note that the drop‐connected network can be considered as a generalization of the drop‐out method. This is because it can highly produce sub‐networks, considering that the number of synaptic weights usually exceeds the number of neurons (Figure S11, Supporting Information).

To evaluate the proposed drop‐connected neural network using the probabilistic SiO*_x_* synaptic barristor, we performed and simulated a shape‐based image classification for several fashion items based on the sigmoidal fitting results of *P*_*Act*_ (i.e., Figure 2h,i and Figure S8, Supporting Information). Figure 3c shows a few examples for three fashion‐item datasets (namely Pullovers, Bags, and Boots) that were used as input learning images.^[^ ^44^ ^]^ For each fashion item, 6,000 learning images and 1,000 inference images were included, each comprised of 28 × 28 grayscale pixels. Figure 3d shows a schematic diagram of the single drop‐connected network containing 784 pre‐neurons (*i* = 1, 2, 3,…784) and 3 post‐neurons (*j* = 1, 2, and 3), together with the TLN functions (gray region). Note that the conductance difference between two neighboring cells can be defined as a single synaptic weight (*W*_*i,j*_ ≡ *G*_*i,j*_ *^+^* − *G*_*i,j*_ *^−^*). However, unlike the conventional backpropagation learning rule (BP), some synapses are partially updated at *P*_*Act*,_ without feedback, such as the derivative of the cost function. Further, the global *V*_*TLN*_ can readily lead to a designated *P*_*Act*_ value. During the learning process, these properties could mitigate circuit overheads in terms of time and energy consumption. The detailed processes for one learning epoch, along with a flow chart of the drop‐connected algorithm, are described in Figure S12 (Supporting Information). Figures 3e,f show part of a drop‐connected network configuration based on the SiO*_x_* barristor array and the stochastic cell activations controlled by the *V*_*TLN*_ and *V*_*SET*_ (see Figure S13, Supporting Information). As shown in Figure 3e, the identical *V*_*SET*_ pulses of *V*_*Pre*_ = 2 V and *V*_*Post*_ = −2 V with *T* = 1 µs were applied to the 16 cells of the selected column line with various *V*_*TLN*_ values. Prior to the programming, all 16 cells were initialized into the “0” state via a *V*_*RESET*_ pulse. Figure 3f shows the experimental *I*_*PSC*_ histograms of the selected 16 cells at *V*_*READ*_ = 1 V as a function of *V*_*TLN*_ after the completion of *V*_*SET*_ programming. As *V*_*TLN*_ changes from −20 to +20 V, the activated cell number of the drop‐connected neural network can be significantly increased from 0 to 16, thus indicating an increase in *P*_*Act*_ from 0 to 1.0. The changeable drop‐connections can entirely determine the output current sum of the selected column line (∑*I*_*PSC*_) based on the vector‐matrix multiplication (the right of Figure 3f), and a higher *P*_*Act*_ can generate a larger ∑*I*_*PSC*_. Therefore, these results verify that the global *V*_*TLN*_ can readily facilitate the probabilistic weight updating in the flexible neural network.

**Figure 4:**
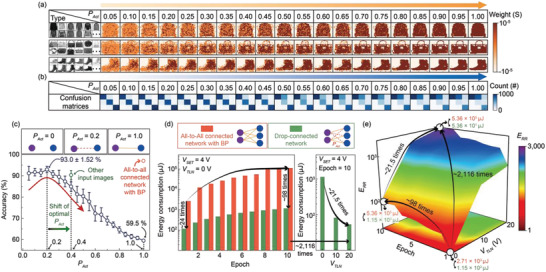
The recognition and energy consumption simulation results for fashion item images. a) Visualization of distribution maps for the updated weight values (S) corresponding to each input fashion‐item dataset following the learning process of the drop‐connected network for various *P*_*Act*_ values ranging from 0 to 1.0. b) The confusion matrices classified from the difference between the targeted and inferred images according to the various *P*_*Act*_ values ranging from 0 to 1.0. c) The average recognition accuracies of the fashion‐item patterns as a function of *P*_*Act*_. The top panels show the synaptic connection for *P*_*Act*_ values of 0, 0.2, and 1.0. The recognition accuracies are found to be ≈93.0 ± 1.52 % and ≈59.5 % for *P*_*Act*_ = 0.2 and 1.0, respectively (blues). For *P*_*Act*_ = 0.2, the estimated recognition accuracy is comparable to the all‐to‐all connected network with BP (reds) (≈97.4 %). Note that the optimal *P*_*Act*_ changes from 0.2 to 0.4 in the case of other input images (T‐shirts, Trousers, and Sneakers), as shown in the green. d) Comparison of the network‐level energy consumption the learning process for the all‐to‐all connected and the drop‐connected neural network. The drop‐connected network can reduce the learning energy by ≈98 times without the application of *V*_*TLN*_ (left). By increasing *V*_*TLN*_ from 0 to 20 V, the learning energy of the drop‐connected network can be further decreased by ≈21.5 times, leading to a more efficient learning process as compared to the all‐to‐all connected network with BP (≈2,116 times better in total) (right). e) Contour plots of the estimated *E*_*RR*_ as functions of *V*_*TLN*_ and epoch. As indicated by the black arrows, *E*_*RR*_ is observed to significantly increase when *V*_*TLN*_ and epoch increase (up to ≈2,116 times). Note that the black circles indicate the detailed energy value consumed at each *V*_*TLN*_ and epoch. The energy consumptions of the all‐to‐all connected network and drop‐connected network is represented in red and green colors, respectively.

**Figure** **4** shows the simulation results for recognition of the above‐mentioned fashion‐item patterns based on the drop‐connected neural network, comprising gate‐tunable and probabilistic SiO*_x_* synaptic barristors according to *P*_*Act*_. Based on experimental *P*_*Act*_ values ranging from 0 to 1.0, we visualized the distribution maps of the updated synaptic weights (*W*_*i,j*_) for each input fashion‐item dataset after completion of the learning process (Figure 4a). Interestingly, the shape of each distribution map is observed to resemble its corresponding input image more closely as *P*_*Act*_ increases. Further, as *P*_*Act*_ approaches 1.0, each cell in the drop‐connected network is more likely to be deterministically updated to either the “0” or “1” state during the learning stage. Figure 4b shows the confusion matrices derived from the classification results between the inferred and targeted images (number of inference images = 1,000) for various *P*_*Act*_ values. The blue saturation of only diagonal tiles in the matrices indicates correct recognition of all inference images, and the diagonal tiles are observed to be most saturated (i.e., the highest recognition accuracy is achieved) when *P*_*Act*_ = 0.2. Indeed, switching certainty (i.e., *P*_*Act*_ = 1.0) during the *W*_*i,j*_ update may cause forgetting (erasing) of a former learned image when the next input image is delivered to the network. Therefore, there is a large possibility of image recognition failure during the inference stage (Figure S14, Supporting Information). Conversely, a moderate uncertainty (i.e., *P*_*Act*_ = 0.2) during the *W*_*i,j*_ update can help the network learn meaningful and common features among different pixels for each fashion input image. In other words, it can have a positive effect on the recognition accuracy for the fashion‐item images. Figure 4c presents the average recognition accuracy after 10 epochs as a function of the *P*_*Act*_, where one epoch indicates one round of the learning process using a dataset of 18,000 learning images. Note that the recognition accuracy were statistically evaluated based on the 10 trials. The maximum recognition accuracy is observed to be ≈93.0 ± 1.5 % at *P*_*Act*_ = 0.2, but it decreases to 59.5 % at *P*_*Act*_ = 1.0. Moreover, the variation in the recognition accuracy is observed to decrease as *P*_*Act*_ increases, indicating the transition from probabilistic to deterministic *W*_*i,j*_ updates. These are consistent with the results of Figure 4a,b. Notably, the optimal *P*_*Act*_ and number of *W*_*i,j*_ updates required for the highest accuracy could be varied when using various kinds of target images due to the intrinsic uncertainty of the probabilistic update process. As indicated by the green in Figure 4c, the optimal *P*_*Act*_ changes from 0.2 to 0.4 when other input images such as T‐shirts, Trousers, and Sneakers are used, in spite of the same network configuration being used (Figure S15, Supporting Information). As *P*_*Act*_ can be readily adjusted to its optimal value for any given task via electrostatic gating, we believe that the proposed gate‐tunable and probabilistic SiO*_x_* synaptic barristor crossbar array is appropriate for the suggested drop‐connected network. Further, the drop‐connected network based on the probabilistic SiO*_x_* synaptic cells exhibits the acceptable recognition accuracy, as compared to the all‐to‐all connected network with the BP (red circle in Figure 4c). This is because the principal features and information of the input dataset can be effectively learned by the optimal *P*_*Act*_ during the *W*_*i,j*_ updates, thus inducing the sub‐network effect and preventing co‐adaptation (Figure S10, Supporting Information).

Figure 4d exhibits the comparison of the energy consumptions at the network level between the all‐to‐all connected (red box) and the drop‐connected network (green box) as a function of learning epoch. For comparison, we assumed that the all‐to‐all connected network comprises typical deterministic analog synapses, while the drop‐connected network comprises the gate‐tunable and probabilistic SiO*_x_* synaptic barristors with *P*_*Act*_ = 0.2 at *V*_*TLN*_ = 0 V (see, the results of Figure S8 and Table S4, Supporting Information). The number of intermediate states of the analog synapse for the all‐to‐all connected network was assumed to be 6 bit, which has been reported as the minimum bit number of analog synapse required for the learning process. In addition to this, each state was set to be in the range between the “0” and “1” conductance values of the probabilistic SiO*_x_* synaptic cells and was programmed at the same electrical input for an unbiased comparison. The two networks were then set to learn via the typical BP and drop‐connected learning rules, respectively. As shown in the left of Figure 4d, when epoch increases from 1 to 10, the drop‐connected network consumes a significantly lower learning energy than the all‐to‐all connected network (≈98 times at 10 epoch). This is primarily because ≈80 % of synaptic connections are iteratively excluded at *P*_*Act*_ = 0.2 so as not to update during the learning process, thus further reducing the energy consumption. Note that the energy consumption at network‐level was estimated from the energy sum of individual synaptic cell that is iteratively updated during learning process (Experimental Section). Moreover, an increase in *V*_*TLN*_ from 0 to +20 V is observed to further decrease the consumed energy by ≈21.5 times, thereby resulting in a total reduction of ≈2,116 times compared to that of the all‐to‐all connected network (Figure 4d,e). Clearly, as shown in Figure 4e, the energy reduction rate (*E*_*RR*_) at 10 epoch significantly increase from ≈98 times to ≈2,116 times as the *V*_*TLN*_ is increased from 0 to 20 V. The *E*_*RR*_ is defined as the reduced energy ratio of drop‐connected network to the all‐to‐all connected one. Note that the electrostatic gating energy in Figure 4d,e was considered based on the leakage current and the capacitive charging energy according to the *V*_*TLN*_ values (Experimental Section). These results verify that the aim of gating in this junction architecture is to efficiently update the synaptic cell and to modulate *P*_*Act*_ on the given task. As a result, we believe that the suggested drop‐connected network, based on gate‐tunable and probabilistic SiO*_x_* synaptic barristor crossbar array, is an another route toward the artificial intelligence applications in the future edge computing paradigm which inevitably requires the low‐energy consuming and simple systems.

## 3 Conclusion

In summary, we presented a gate‐tunable and probabilistic SiO*_x_* synaptic barristor crossbar array and a drop‐connected neural network that can mimic the energy‐efficient sparse activity of a biological neural network. The switching‐transition characteristic between two different Si phases can be actively modulated via electrostatic gating in order to mimic the threshold‐tunable and probabilistic synaptic functionalities of a rod‐to‐rod bipolar synapse. Compared with the conventional all‐to‐all connected network based on typical deterministic synapses, the drop‐connected neural network comprising the probabilistic SiO*_x_* synaptic barristors with *P*_*Act*_ = 0.2 can exhibit superior energy and learning efficiency for recognizing fashion‐item images. We believe that the probabilistic SET nature of the SiO*_x_* synaptic barristor, its low‐power programming driven by electrostatic gating, and the probabilistic synaptic updating process based on the biological sparse features enable energy‐ and learning‐efficient neuromorphic computation.

## 4 Experimental Section

### Fabrication of the Gate‐Tunable and Probabilistic SiO_x_ Synaptic Barristor Crossbar Array

The gate‐tunable and probabilistic SiO*_x_* synaptic barristor crossbar array on a highly doped p‐type (100) Si wafer (1.2 × 1.2 cm) covered with thermally grown 285‐nm‐thick SiO_2_ was fabricated. First, the SiO_2_/Si substrate was cleaned by sequential ultrasonication in acetone, isopropyl alcohol, and deionized water for 3 min each. Then, a monolayer graphene was transferred to the top side of the substrate via a standard chemical vapor deposition (CVD) method using H_2_ and CH_4_ at 1000 °C. To form 16 source lines with a width of 20 µm for the post‐neurons, a photoresist (AZ5214E) was spin‐coated and patterned using standard UV‐mask photolithography and reactive ion etching (RIE). Subsequently, 16 drain lines (Pd (50 nm)/SiO*_x_* (60 nm)) with a width of 20 µm for the pre‐neurons were patterned and deposited perpendicularly across the post‐neuron lines under the same photolithography and e‐beam evaporator.

### Electrical Characterization

A semiconductor parameter analyzer (4155C, Agilent), pulse generator (81104A, Keysight), and low‐leakage switch mainframe (E5250A, Keysight) with a vacuum probe station (working pressure <10^−5^ torr) were utilized to investigate and characterize the *I*_*PSC*_ *–V*_*Pre*_ switching characteristics, the *V*_*TLN*_ tunability, the *P*_*Act*_ behaviors, and the retention capability of the gate‐tunable and probabilistic SiO*_x_* synaptic barristor crossbar array.

### Estimation of Energy Consumption at the Network Level during the Learning Process

To estimate the energy consumption at the network‐level, *G*_*i,j*_ *^±^* after every updating process were iteratively extracted to obtain all the *I*_*PSC*_ values as *I*_*PSC*_ = *V*_*READ*_ × *G*_*i,j*_ *^±^*. The energy consumption of individual synaptic cells at every learning steps was then obtained from the following equation, *V*_*SET*_ × *I*_*PSC*_ × *T*. After completion of all the learning epoch, the consuming‐energy sum of all the synaptic cells was extracted. In the case of the drop‐connected network, however, all synaptic cells were probabilistically updated during the SET programming depending on the *P*_*Act*_. Due to the extremely low *I*_*PSC*_ (“0” state), it was acceptable to exclude all the non‐updated cells when calculating the energy consumption at the network‐level. The *V*_*SET*_ was set to 4 V with *T* = 1.02 µs for *P*_*Act*_ = 0.2 (Figure S8 and Table S4, Supporting Information). For an unbiased comparison, the all‐to‐all connected network with the deterministic 6‐bit synapses was assumed to achieve *P*_*Act*_ = 1.0 by application of the same *V*_*SET*_ and *T* values. To consider the gating energy induced by the *V*_*TLN*_, the capacitance value of the graphene/SiO_2_/P^++^‐Si junction structure was measured as ≈1.18 nF, which is almost identical to the theoretical capacitance value of the 285 nm‐thick SiO_2_ (≈1.21 nF). Based on the capacitance values with a cell area of 400 µm^2^, the capacitive charging energy was evaluated by $\frac{1}{2}CV_{TLN}^{2}$ during the SET‐switching transition. Hence, for *V*_*TLN*_ = 0, +10, and +20 V, the gating energies were estimated to be 0, 2.36, and 9.44 pJ, respectively. As the gate leakage current (*I*_*TLN*_) was as much as ≈10^5^ times lower than the *I*_*PSC*_, the gating energy induced by the leakage current was almost negligible.

## Conflict of Interest

The authors declare no conflict of interest.

## Supporting information

Supporting Information

## Data Availability

All the data and the findings of this paper are available within the article and its Supporting Information or from the corresponding author upon reasonable request.
