# Supplementary material for: Flexible Neural Network Realized by the Probabilistic SiO x Memristive Synaptic Array for Energy‐Efficient Image Learning
Source: Adv Sci (Weinh). 2022 Feb 16;9(11):2104773. doi: 10.1002/advs.202104773 (PMC9009121; doi:10.1002/advs.202104773)
Supplement: Supplementary file 1 — Supporting Information [file ADVS-9-2104773-s001.pdf]

ADVANCED SCIENCE
Open Access

Supporting Information

for *Adv. Sci.*, DOI 10.1002/advs.202104773

Flexible Neural Network Realized by the Probabilistic SiO_x Memristive Synaptic Array for Energy-Efficient Image Learning

*Sanghyeon Choi, Jingon Jang, Min Seob Kim, Nam Dong Kim, Jeehyun Kwag and Gunuk Wang**

ADVANCED SCIENCE
Open Access

Supporting Information

for *Adv. Sci.,* DOI: 10.1002/advs.202104773

Flexible neural network realized by the probabilistic SiO*x* memristive synaptic array for energy-efficient image learning

*Sanghyeon Choi, Jingon Jang, Min Seob Kim, Nam Dong Kim, Jeehyun Kwag and Gunuk Wang**

Supporting Information for

Flexible neural network realized by the probabilistic SiO_x memristive synaptic array for energy-efficient image learning

Sanghyeon Choi, Jingon Jang, Min Seob Kim, Nam Dong Kim, Jeehyun Kwag and Gunuk Wang[*]

Sanghyeon Choi, Jingon Jang, and Prof. Gunuk Wang*
KU-KIST Graduate School of Converging Science and Technology, Korea University, 145 Anam-ro, Seongbuk-gu, Seoul, 02841, Republic of Korea

Min Seob Kim, Dr. Nam Dong Kim
Institute of Advanced Composite Materials, Korea Institute of Science and Technology, 92 Chudong-ro, Bongdong-eup, Wanju-gun, Jeollabuk-do 55324, Republic of Korea

Prof. Jeehyun Kwag
Department of Brain and Cognitive Engineering, Korea University, Seoul, 02841, Republic of Korea

Prof. Gunuk Wang*
Department of Integrative Energy Engineering, Korea University, 145 Anam-ro, Seongbuk-gu, Seoul, 02841, Republic of Korea

**Corresponding e-mail:* gunukwang@korea.ac.kr

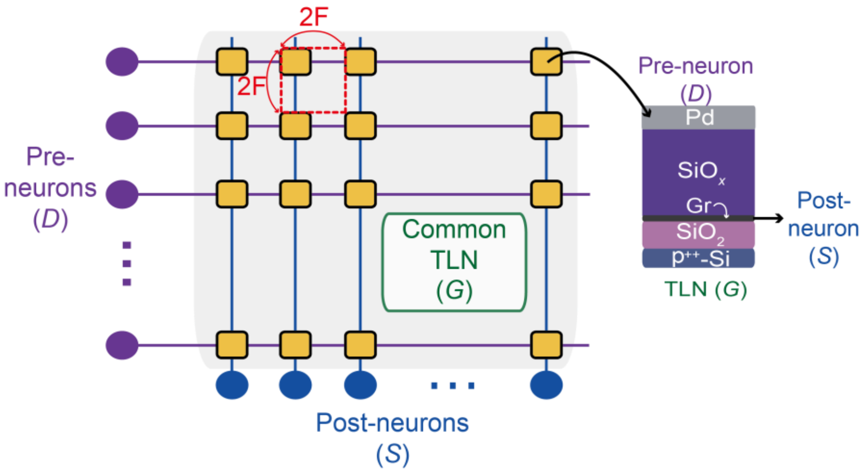

Figure S1. Schematic diagram of the crossbar array structure consisting of vertically integrated SiO_x memristive synaptic barristor (inset). The vertical form of the fabricated synaptic cells enables the $4F^2$ footprint, leading to the highly-dense integration like two-terminal memristors. Note that we used p^{++}–Si substrate as a common gate, thus the crossbar array structure employing drain (pre-neuron) and source (post-neuron) lines can be formed.

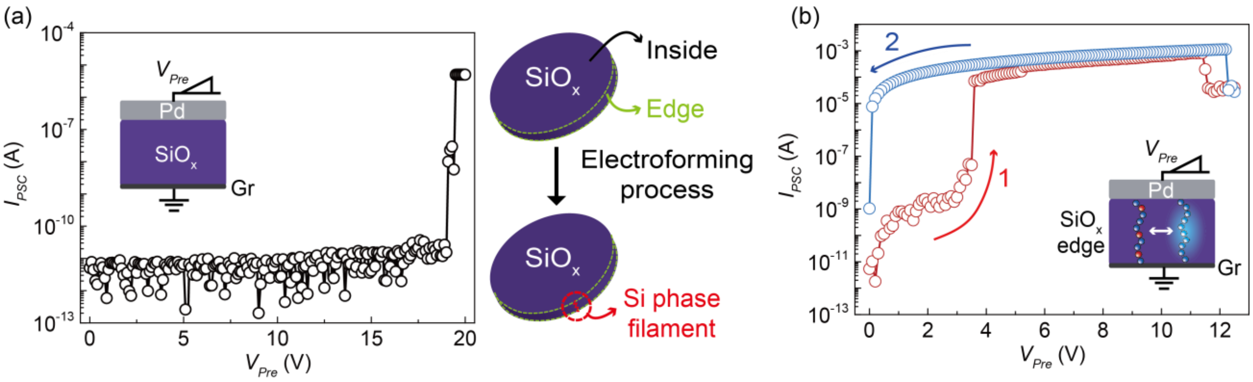

Figure S2. (a) The representative initial I_{PSC}–V_{Pre} curve of the memristor cell of SiO_x synaptic barristor during the electroforming process, together with the operating bias scheme (inset). As shown in the right insets, the electroforming process, which is achieved by the application of V_{Pre} to a certain high value (~20 V), can induce the energetically viable redox transformation from SiO_x to Si phases (red line) at the localized edge of SiO_x matrix (the green dotted region). Diverse evidences, such as TEM analysis,[S1,S2] electroluminescent (EL) result,[S3] and electrical characteristics,[S2] have demonstrated the formation of Si phase filament at the SiO_x edge. Note that the V_{Pre} to generate a sudden increase in I_{PSC} is regarded as the electroforming voltage. (b) The representative I_{PSC}–V_{Pre} switching curve of the memristor cell of SiO_x synaptic barristor after the completion of electroforming process. Note that the sweep direction is operated following the number 1 and 2. The inset illustrates the phase transition of the Si filament corresponding to the switching events where the left and right filaments represent the "0" (OFF) and "1" (ON) states, respectively. Note that blue and red circles represent Si-NC and α-Si, respectively.

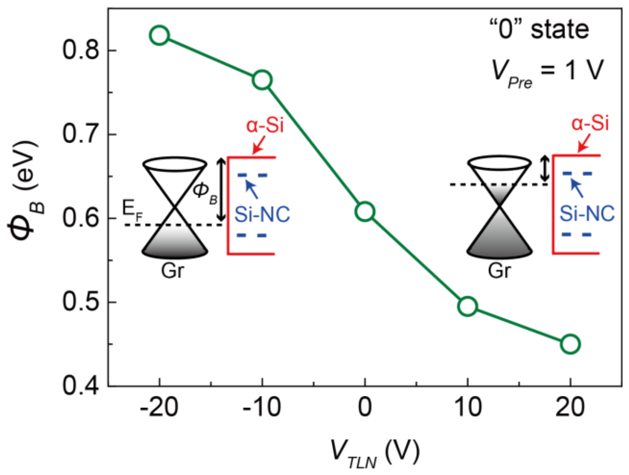

Figure S3. Plot of the Φ_B values in "0" state at V_{Pre} = 1 V as a function of V_{TLN}. The insets show the Φ_B (black arrows) at the Si phases/Gr interface according to the location of E_F of the Gr that can be modulated V_{TLN}.

The Schottky barrier height (Φ_B) formed at the Si phase filament/Gr interface can be quantitatively estimated by using the charge transport theory of graphene[S4-S6]:

$$\Phi_B = k_B T \ln\left(\frac{A^* T^3}{J_{PSC}}\right) - E_F$$

$$\text{where, } E_F = \hbar v_f \sqrt{\pi \frac{C_{TLN}(V_{TLN} - V_{Dirac})}{e}}$$

Note that k_B is Boltzmann constant; T is the temperature; A^* is the effective Richardson constant; J_{PSC} is the current density; E_F is the Fermi level of graphene; $\hbar$ is the Planck constant; v_f is Fermi velocity; C_{TLN} is the gate capacitance density, and V_{Dirac} is the Dirac point of graphene; e is the electron charge.

Based on this equation, we quantitatively estimated the Φ_B at the Si phases/Gr interface in the "0" state at V_{Pre} = 1 V as a function of V_{TLN} from –20 V to 20 V (Figure S3). As shown in Figure S3, the Φ_B at the Si phases/Gr interface is decreased from 0.82 to 0.45 eV when increasing V_{TLN} from -20 to +20 V. This is because the E_F of Gr can be electrostatically modulated via the application of V_{TLN}.[S7] As a result of this effect, V_{TLN} changes the effective electric field required for crystallization in α-Si phases, thereby enabling the different V_{SET}. Note that the C_{TLN} value was electrically characterized in the Gr/SiO_2/p^{++}-Si junction, which was found to be ~11.8 nF/cm^2. Also, the V_{Dirac} value was observed to be -1 V in the Pd/graphene/Pd lateral junction at V_{Pre} = 1 V as a function of V_{TLN} (Figure S4). Other parameters are provided in Table S1.

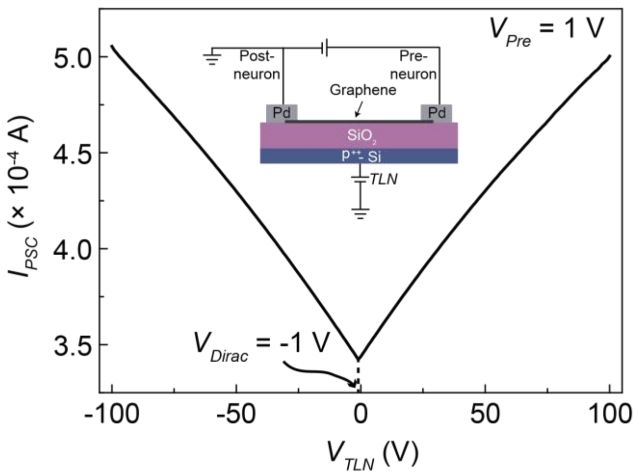

Figure S4. Plot of I_{PSC}–V_{TLN} curve for the Pd/Gr/Pd lateral junction at V_{Pre} = 1 V. V_{Dirac} is found to be -1 V. The inset illustrates a schematic diagram of the Pd/Gr/Pd lateral junction on the SiO_2/p^{++}–Si.

k_B (J·K^{-1})	T (K)	A^* (A·cm^{-2}·K^{-3})	$\hbar$ (J·s)	v_f (cm/s)	e (C)
1.38×10^{-23}	300	0.01158[S5]	1.05×10^{-34}	1.15×10^{8}[S7]	1.60×10^{-19}

Table S1. The used parameters for the quantitative estimation of Φ_B.

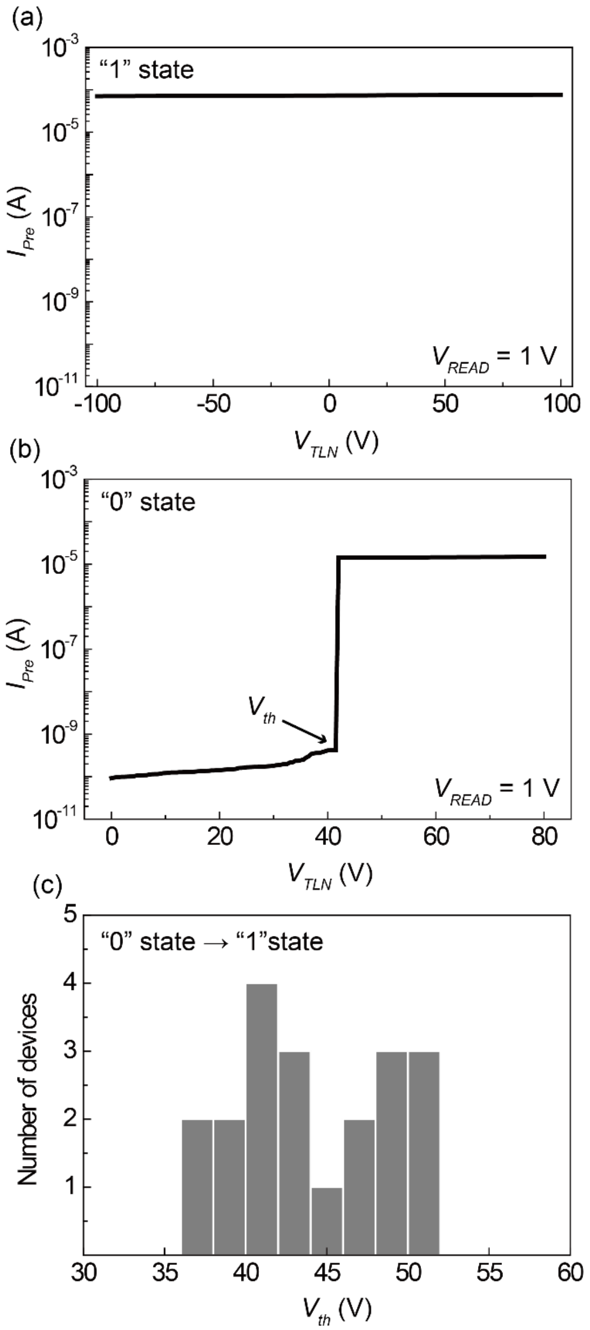

Figure S5. (a, b) Plots of the I_{PSC} values at V_{READ} = 1 V as a function of V_{TLN} with respect to "1" and "0" states, respectively. Because the dirac cone shape of graphene requires a lot of gate bias for electron doping at V_{READ} = 1 V, the SET–switching transition do not occur until V_{TLN} passes the very high value (> 37 V). This threshold voltage is defiend as V_{th}. Considering the used V_{TLN} values ranging from -20 V to 20 V, V_{READ} of 1 V can be considered a reasonable value. (c) Statistical histograms of V_{th} values for the selected 20 cells in the array. The statistical V_{th} was found to be 44.20 ± 4.62 V.

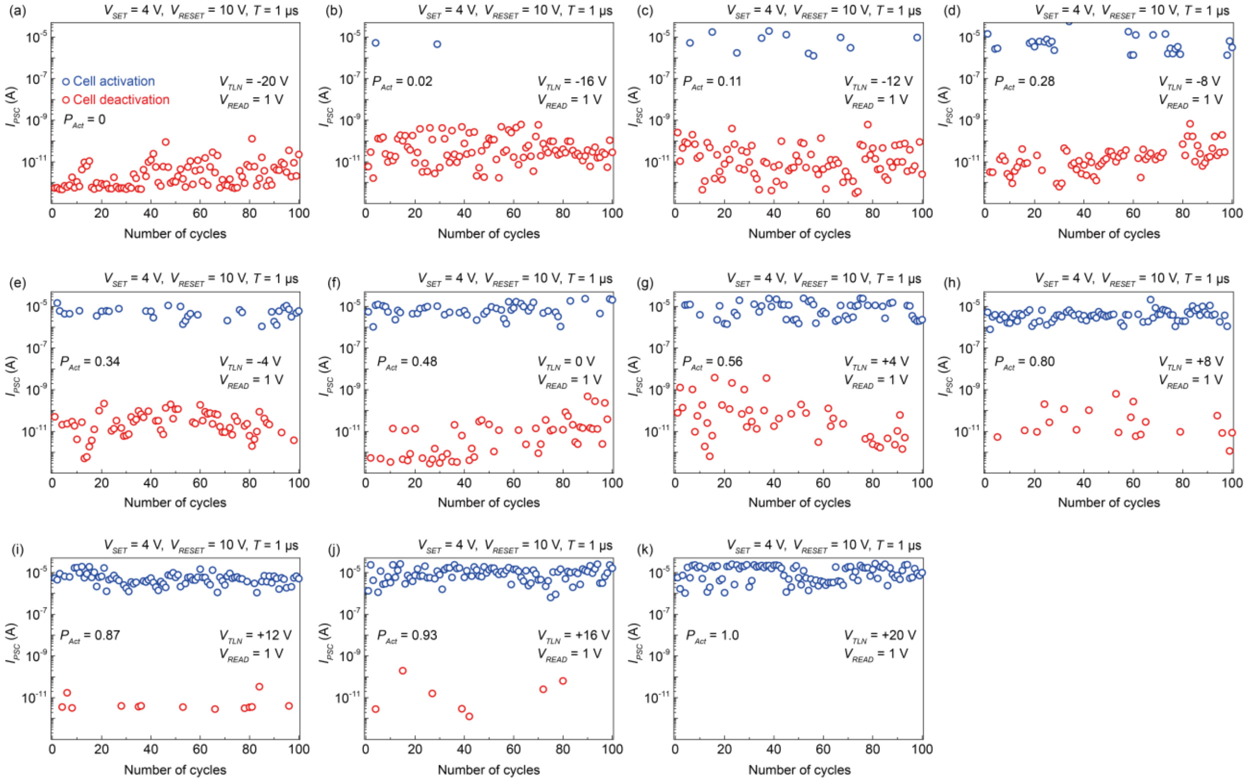

Figure S6. (a-k) Plots of the I_{PSC} values at V_{READ} = 1 V after application of a V_{SET} pulse during 100 consecutive cycles at various V_{TLN} values ranging from -20 V to +20 V. One cycle consisted of V_{READ} = 1 V, V_{SET} = 4 V, V_{READ} = 1 V, and V_{RESET} = 10 V for T = 1 μs, and V_{TLN} was set to (a) -20, (b) -16, (c) -12, (d) -8, (e) -4, (f) 0, (g) +4, (h) +8, (i) +12, (j) +16, and (k) +20 V. Cell activation (final I_{PSC}/initial I_{PSC} >10^5) and deactivation (final I_{PSC}/initial I_{PSC} <10^5) are represented as blue and red circles, respectively. The P_{Act} is varied from 0 to 1.0 depending on the V_{TLN}. Note that the initial and final I_{PSC} values were measured at the initial and final V_{READ} of one cycle.

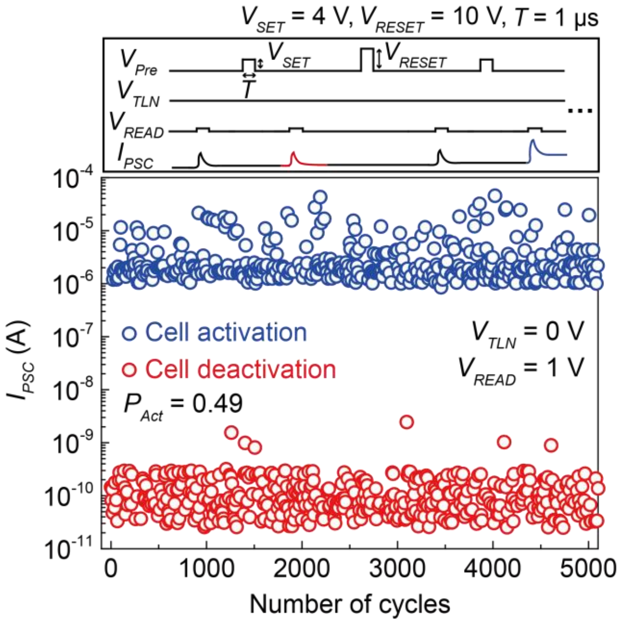

Figure S7. A plot of the I_{PSC} values at V_{READ} = 1 V after application of a V_{SET} pulse during the more than 5,000 cycles with respect to V_{TLN} = 0 V. As shown in the top panel, one cycle consisted of V_{READ} = 1 V, V_{SET} = 4 V, V_{READ} = 1 V, and V_{RESET} = 10 V for T = 1 μs, and V_{TLN} was set to 0 V. The fabricated SiO_x memristive synaptic barristor is observed to endure the consecutive incoming input pulses more than 5,000 cycles (i.e., > 20,000 pulse in total) with P_{Act} = 0.49.

100 cycles, T = 1 μs

Parameters / V_{SET}	a	b	c
3.5 V	1.19	0.12	8.59
4.0 V	1.05	0.15	1.44
4.5 V	1.03	0.16	-1.80

20 cells, T = 1 μs

Parameters / V_{SET}	a	b	c
	1.06	0.15	6.61
4.0 V	0.98	0.17	0.28
4.5 V	1.03	-0.19	0.18

Table S2. Summary of fitting parameters of the sigmodial fitting curves at V_{SET} = 3.5 V, 4.0 V, and 4.5 V for T = 1 μs as a function of V_{TLN} with respect to Figure 2h (upper, for 100 cycles) and Figure 2i (lower, for 20 cells).

100 cycles, T = 1 μs

V_{SET} \ V_{TLN}	-20 V	-16 V	-12 V	-8 V	-4 V	0 V	4 V	8 V	12 V	16 V	20 V
3.5 V	0 / 100	0 / 100	4 / 100	15 / 100	21 / 100	32 / 100	49 / 100	55 / 100	69 / 100	81 / 100	100 / 100
4.0 V	0 / 100	2 / 100	11 / 100	28 / 100	34 / 100	48 / 100	56 / 100	80 / 100	87 / 100	93 / 100	100 / 100
4.5 V	0 / 100	6 / 100	16/ 100	30/ 100	46 / 100	56 / 100	73 / 100	86 / 100	91 / 100	100 / 100	100 / 100

20 cells, T = 1 μs

V_{SET} \ V_{TLN}	-20 V	-16 V	-12 V	-8 V	-4 V	0 V	4 V	8 V	12 V	16 V	20 V
3.5 V	0 / 20	0 / 20	0 / 20	1 / 20	3 / 20	8 / 20	9 / 20	12 / 20	13 / 20	16 / 20	20 / 20
4.0 V	0 / 20	0 / 20	2 / 20	3 / 20	7 / 20	11 / 20	12 / 20	15 / 20	17 / 20	18 / 20	20 / 20
4.5 V	0 / 20	1 / 20	2 / 20	4 / 20	7 / 20	11 / 20	14 / 20	16 / 20	19 / 20	20 / 20	20 / 20

Table S3. All the number of cell activation at V_{SET} = 3.5 V, 4.0 V, and 4.5 V for T = 1 μs as a function of V_{TLN} with respect to the 100 cycles (upper) and 20 cells (lower).

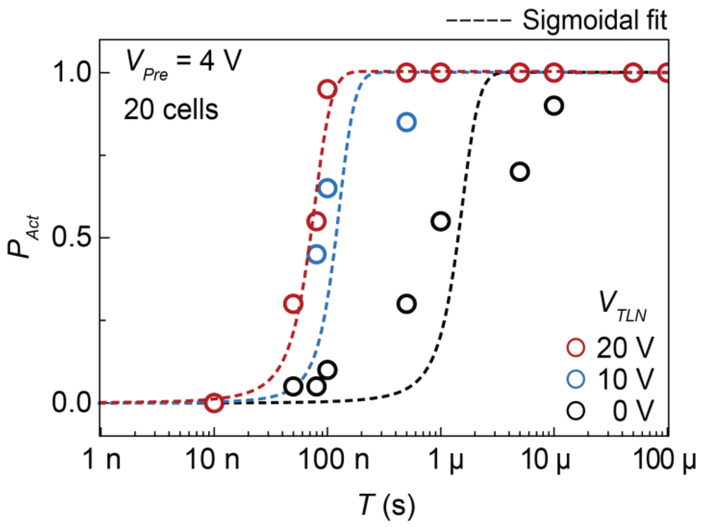

Figure S8. Plots of P_{Act} values at V_{TLN} = 0 V, 10 V, and 20 V as a function of T from 10 ns to 100 μs. The V_{SET} was set to 4 V. All the plots also follow the sigmoidal curves (dotted lines), and are mathmatically expressed as $P_{Act} = a / (1 + \exp(-b \times (T-c)))$. The fitting parameters a, b, and c are providied as below.

V_{TLN} \ Parameters	a	b	c
0 V	1.00	2.99×10^{6}	1.46×10^{-6}
10 V	1.00	5.26×10^{7}	9.82×10^{-8}
20 V	1.00	5.96×10^{7}	7.02×10^{-8}

Table S4. Summary of fitting parameters of the sigmodial fitting curves at V_{SET} = 4.0 V and V_{TLN} = 0, 10, and 20 V as a function of T for 20 cells.

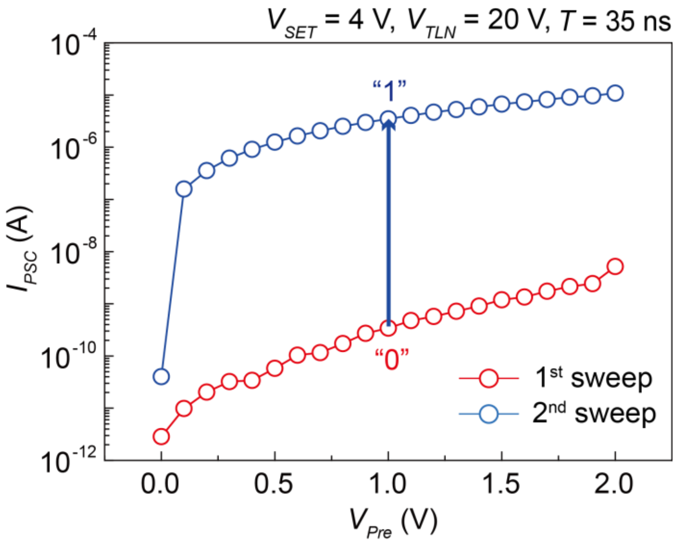

Figure S9. The I_{PSC}–V_{Pre} curves swept from 0 to 2 V before and after the SET–switching transition with the V_{SET} = 4 V and V_{TLN} = 20 V for T = 35 ns. The vertical blue arrow indicates the direction of SET-switching transition. The rising edge and falling edge of the pulses are 8 ns.

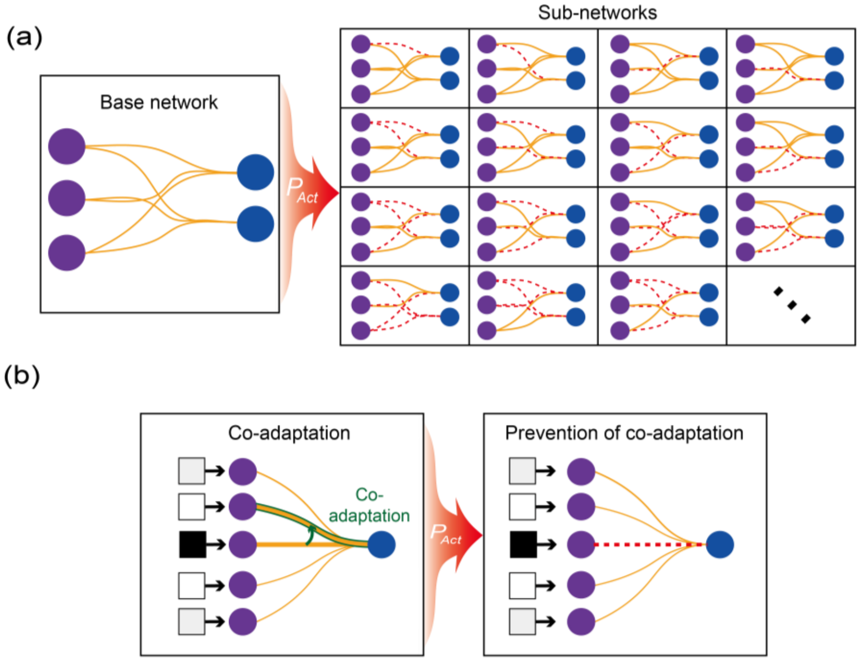

Figure S10. (a) Ensemble effect. Base network can have several sub-networks determined by P_{Act}. In the sub-networks, synaptic connections are randomly selected (yellow lines) and dropped (red dotted lines) based on P_{Act} whenever the drop-connected network learns each input image, which can result in a wide set of sub-networks. Consequently, these sub-networks can induce the

ensemble effect.[S8] (b) Prevention of co-adaption. Conventional all-to-all connected networks might suffer from co-adaptation by abnormal synaptic connections. Conversely, the drop-connected network is afforded some degree of immunity by P_{Act}.[S9]

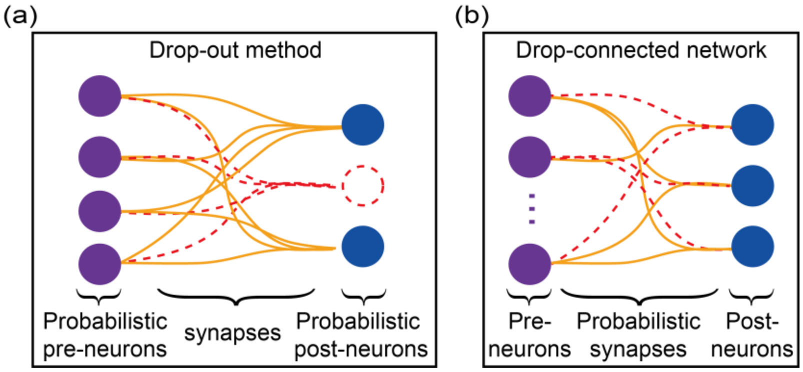

Figure S11. Schematic diagrams of (a) the drop-out method and (b) the drop-connected network. The neurons are randomly dropped for the drop-out method. The synapses are randomly dropped for the drop-connected network. The red dotted lines and circles indicate the dropped synaptic weights and neurons, respectively. Notably, the drop-connected network can be considered as the generalized approach of the drop-out method due to the higher degree of sparse connectivity.

The traditional drop-out method randomly selects some neurons in each layer, and then they are set to 0 along with their all-synaptic connections during the learning stage (Figure S11a). However, for the proposed drop-connected network, certain synaptic weights are randomly selected and updated by designating a probability P_{Act}, while the drop-connection occurs at 1-P_{Act}. (Figure S11b). Briefly, drop-connected network randomly drops the synaptic weights, whereas drop-out method drops the neurons. They are all effective for learning neural networks based on the ensemble effect and prevention of co-adaptation. Notably, the drop-connected network can be regarded as a generalized network configuration of the drop-out method because it can produce even more possible sub-networks through the number of synaptic weights larger than the number of neurons. For example, the drop-connected network can generate a certain sub-network induced by the drop-out method during the learning stage. Hence, the drop-connected network is expected to outperform the drop-out method based on the higher degree of sparse connectivity.[S10]

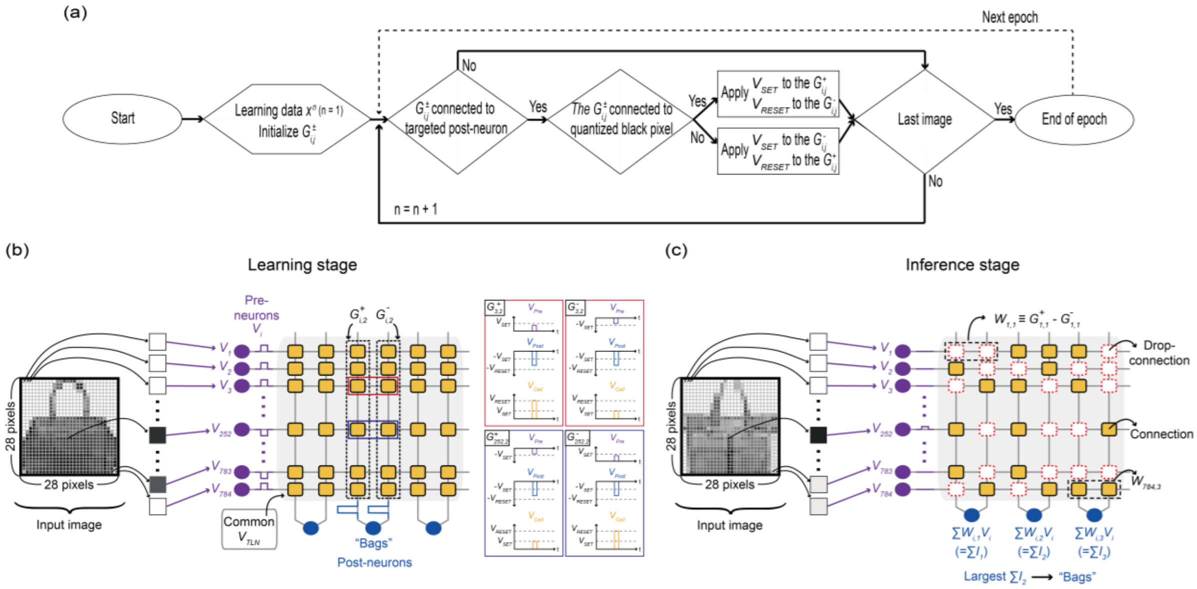

Figure S12. (a) A flow chart for one learning epoch in the drop-connected algorithm. (b,c) Schematic diagrams of the suggested drop-connected network configurations and the programming schemes during (b) the learning and (c) inference stage.

As displayed in Figure S12a, the learning data (X^n) of 18,000 fashion images are used to evaluate the recognition capabilities of the drop-connected neural network. The network initializes all the probabilistic synapses ($G_{i,j}^{\pm}$) to the "0" state, and then individually assigns Pullovers, Bags, and Boots to the 1st–3rd post-neurons, respectively. For example, when a Bag image is fed into the network, each $G_{i,j}^{\pm}$ connected to a targeted post-neuron is individually updated by the programming V_{SET} and V_{RESET} pulse scheme (Figure S12a and S12b). Note that a pair of $G_{i,j}^{\pm}$ is comprised of a single synaptic weight ($W_{i,j}$) (Figure S12b and S12c). The programming scheme is determined by whether $G_{i,j}^{\pm}$ is connected to the quantized black pixel of the input image. In other words, during the learning stage, the different pulse schemes are applied based on whether the input pixel is closer to black (e.g., V_{Pre} = -3 V and V_{post} = -7 V, namely, V_{SET} = 4 V for T = 1 µs) or white (e.g., V_{Pre} = 3 V and V_{post} = -7 V, namely, V_{RESET} = 10 V for T = 1 µs); however, each $G_{i,j}^{\pm}$ connected to untargeted post-neurons leave as it is (Figure S12a and S12b). Note that additional modulation of P_{Act} can be enabled by the common V_{TLN}. Because the identical V_{TLN} is required to probabilistically update all the cells with a certain P_{Act}, the common V_{TLN} can be considered as an acceptable programming scheme for the drop-connected network configuration. Using these pulse schemes, the synaptic connections in the network are randomly updated according to the P_{Act} value of the SiO_x memristive synaptic barristor (Figure S12a and S12b).

During the inference stage for the Bag images, the input reading voltage (V_i) at a pre-neuron corresponding to a black or white pixel (e.g., 1 or 0 V) is individually scaled by the conductance of a synapse ($G_{i,j}^{\pm}$), and then delivered to a post-neuron in the form of a transmitted post-synaptic current ($\sum(G_{i,j}^{+} - G_{i,j}^{-})V_i = \sum W_{i,j}V_i = \sum I_j$), where the subscripts i and j indicate the ith pre-neuron and j^{th} post-neuron, respectively (Figure S12c). As shown in Figure S12c, if the 2nd post-neuron is

fired with the largest $\sum I_j$ during the inference process, the input test image can be inferred as Bags (Pullovers and Boots are assigned to the 1st and 3rd post-neurons, respectively).

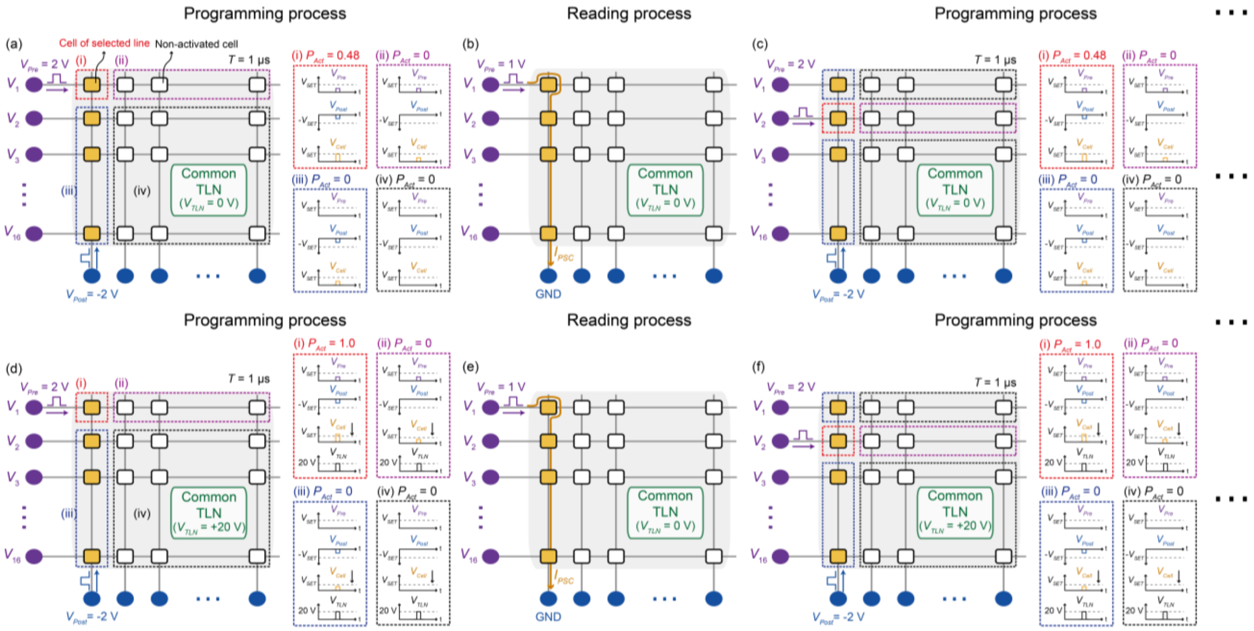

Figure S13. Examples of schematic diagrams of the programming and reading processes during the drop-connection test. The programming voltage pulses of V_{Pre} = 2 V and V_{Post} = -2 V for T = 1 µs were used with (a-c) V_{TLN} = 0 and (d-f) 20 V. V_{READ} = 1 V was used, and V_{TLN} was introduced via the common gating. The yellow and white boxes represent the selected and non-activated cells, respectively. The right dotted boxes are the programming voltage schemes corresponding to (i)-(iv) regions. The red dotted box (i) indicates the selected cell, whereas the purple (ii), blue (iii), and black dotted boxes (iv) indicate the other cells.

As shown in Figure S13, we sequentially programmed and read the 16 cells (represented yellow boxes) located in the leftmost column to avoid the crosstalk signal. Note that other cells located in the unselected columns are non-activated, namely, non-electroformed states (represented white boxes). For example, as shown in Figures S13a-S13c, the cells of the selected column line were individually programmed based on the V_{Pre} = 2 V and V_{post} = -2 V for T = 1 µs with respect to V_{TLN} = 0 V. Thus, V_{SET} = 4 V for T = 1 µs was applied to only the selected cells (red dotted box, (i) in Figures S13a-S13c) in this programming and it is expected to P_{Act} = 0.48. The applied voltages for other cells in (ii)-(iv) fell short of V_{SET} (purple, blue, and black dotted boxes) (P_{Act} = 0). After the programming, each cell was readout at V_{READ} = 1 V. From this way, 16 cells can be individually programmed with P_{Act} = 0.48. Similarly, as shown in Figures S13d-S13f, the selected cells were individually programmed and read at the identical voltage pulses with respect to V_{TLN} = 20 V. This programming scheme can result in P_{Act} = 1.0. Therefore, even if the common gate is used, we can selectively program the selected cells using V_{Pre} and V_{Post} pulse schemes. Note that the programming/reading disturbances were not observed regardless of V_{TLN} value, which might be attributed to the selection pulse scheme and non-activated cells in the array. Therefore, this

programming/reading way enables the operation of the drop-connected network by alleviating the undesired disturbance issue.

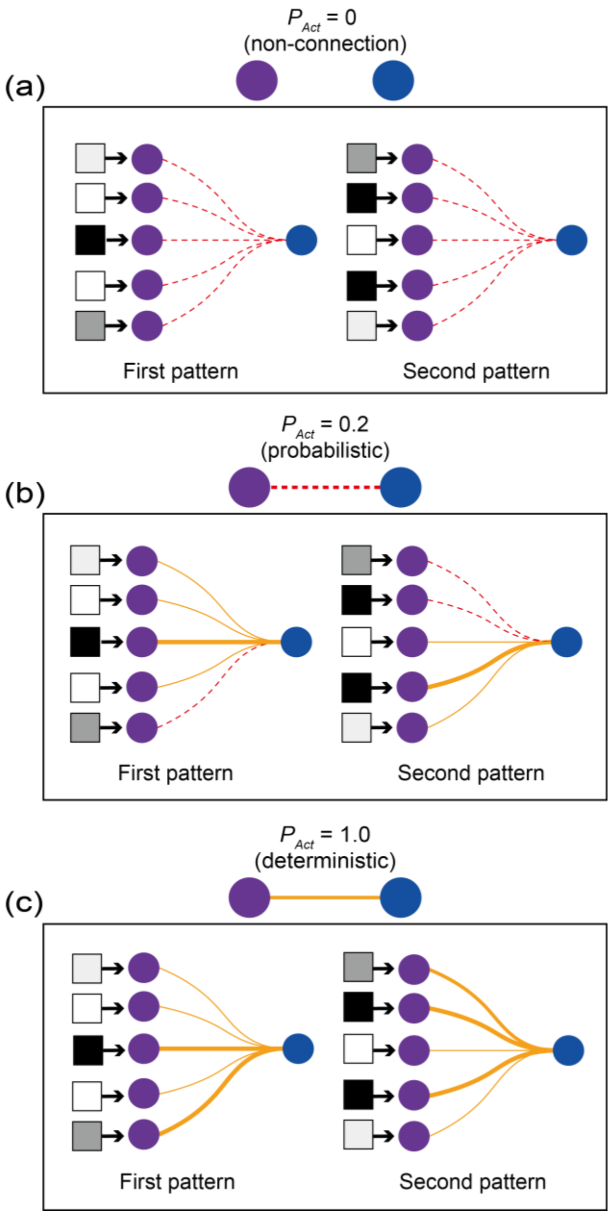

Figure S14. (a-c) Examples of the synaptic connections made during the learning process for the first and second patterns at P_{Act} = (a) 0, (b) 0.2, and (c) 1.0.

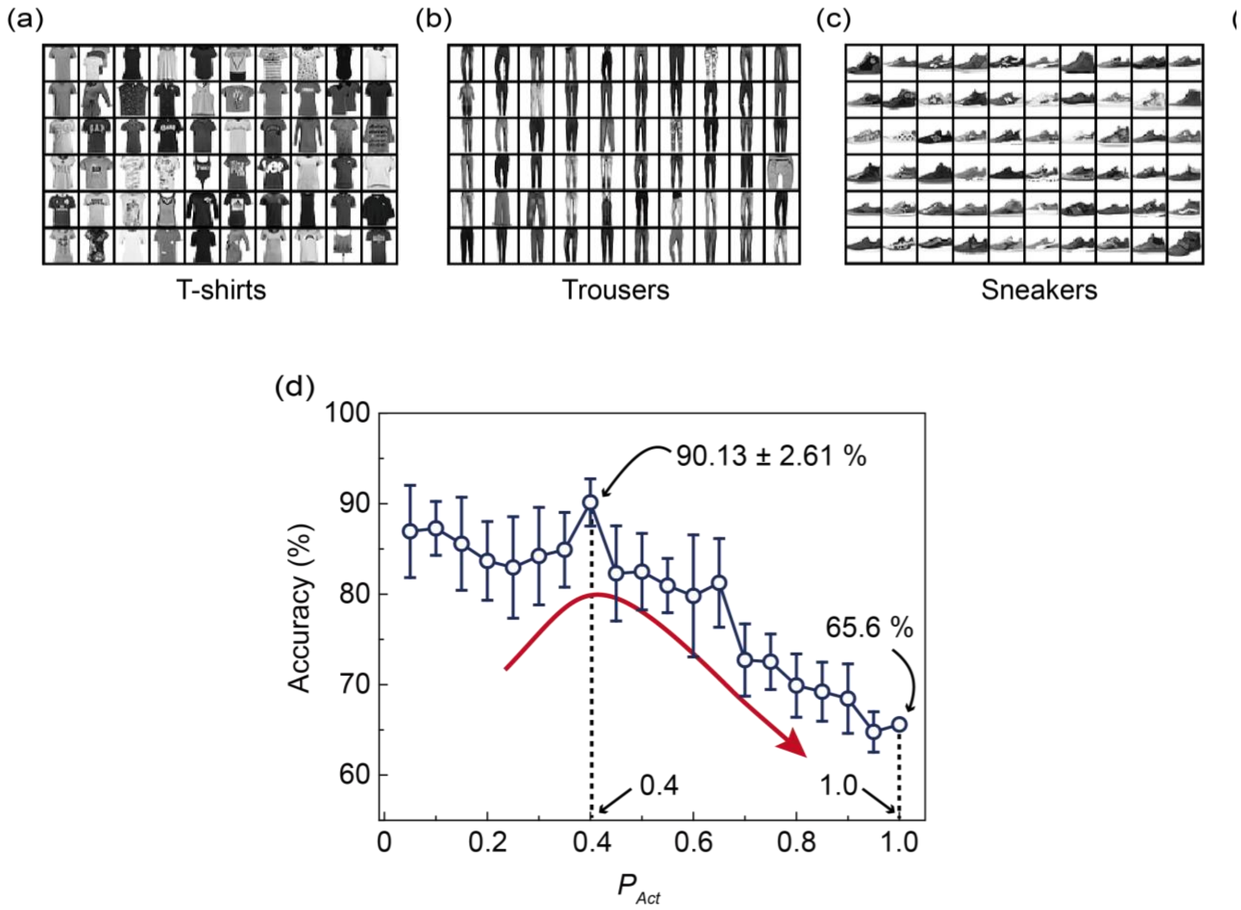

Figure S15. (a-c) Examples of other fashion-item datasets (T-shirts, Trousers, and Sneakers). Each fashion item has 6,000 and 1,000 different image datasets for the learning and inference stages, respectively. (d) The average recognition accuracies of other fashion-item patterns as a function of P_{Act}. The recognition accuracies are found to be ~90.13 ± 2.61 % and ~65.6% for P_{Act} = 0.4 and 1.0, respectively. The optimal P_{Act} value for the high accuracy shifts from 0.2 to 0.4, as the types of input images changes (Figures 4c and S15d).

Reference

[S1] J. Yao, L. Zhong, D. Natelson, J. M. Tour, *Sci. Rep.* **2012**, *2*, 242.

[S2] S. Choi, J.-W. Choi, J. C. Kim, H. Y. Jeong, J. Shin, S. Jang, S. Ham, N. D. Kim, G. Wang, *Nano Energy* **2021**, *84*, 105947.

[S3] C. He, J. Li, X. Wu, P. Chen, J. Zhao, K. Yin, M. Cheng, W. Yang, G. Xie, D. Wang, *Adv. Mater.* **2013**, *25*, 5593.

[S4] S.-J. Liang, L. Ang, *Phy. Rev. Appl.* **2015**, *3*, 014002

[S5] S.-J. Liang W. Hu, A. Di Bartolomeo, S. Adam, L.K. Ang, *IEEE IEDM*, **2016**, 14.14. 11

[S6] S. Rehman, H. Kim, M. F. Khan, J-H. Hur, J. Eom, D.-K. Kim, *J. Alloys Compd.*, **2021**, *855*, 157310.

[S7] Y.-J. Yu, Y. Zhao, S. Ryu, L.E. Brus, K.S. Kim, P. Kim, *Nano Lett*, **2009**, *9*, 3430.

[S8] K. Hara, D. Saitoh, H. Shouno, presented at *the International Conference on Artificial Neural Networks*, Barcelona, Spain, September **2016**.

[S9] G. E. Hinton, N. Srivastava, A. Krizhevsky, I. Sutskever, R. R. Salakhutdinov, (Preprint) arXiv: 1207.0580, submitted: Jul **2012**.

[S10] L. Wan, M. Zeiler, S. Zhang, Y. Le Cun, R. Fergus, presented at *the 30th International Conference on Machine Learning*, Georgia, USA, **2013**.
